# Unraveling fibrinogen-like protein 1’s role in immune regulation

**DOI:** 10.3389/fimmu.2026.1753785

**Published:** 2026-02-18

**Authors:** Fengjia Xi, Rongzeng Liu

**Affiliations:** Department of Immunology, College of Basic Medicine and Forensic Medicine, Henan University of Science and Technology, Luoyang, China

**Keywords:** fgl1, immune regulation, therapeutic target, tumor, autoimmune disease

## Abstract

Fibrinogen-like protein 1 (FGL1) has been recently identified as an emerging novel checkpoint ligand of lymphocyte activation gene-3 (LAG-3) with important immunoregulatory functions. In addition to LAG-3, FGL1 also interacts with bone morphogenetic protein 6 (BMP6), activin receptor-like kinase 5 (ALK5) and other unidentified receptors to perform biological functions. Physiologically, FGL1 restrains intrahepatic immunity and preserves tolerance. Pathologically, FGL1 is frequently upregulated in various tumors and autoimmune diseases and is closely related to the occurrence and development of these diseases. Targeting FGL1 has shown preclinical efficacy in enhancing immunotherapy involving programmed death ligand 1 (PD-L1)/PD-1 checkpoint blockade, inhibiting liver metastasis and relieving autoimmunity without overt hepatotoxicity. In this review, we summarize recent advances in FGL1, focus on the immunoregulatory functions of FGL1, and evaluate its potential as a therapeutic target for immune-related diseases.

## 1 Introduction

Fibrinogen-like protein 1 (FGL1) is a hepatokine that circulates at high concentrations and is conserved across species. It comprises a coiled-coil N-terminus and a fibrinogen-like C-terminal domain that forms homodimers or oligomers, similar to fibrinogen (1, 2). The expression of FGL1 is controlled by cytokine-responsive transcription factors, such as signal transducer and activator 3 (STAT3), hepatocyte nuclear factor-1α (HNF-1α), and CCAAT enhancer-binding protein β (C/EBPβ) (3–5). In addition, posttranslational modifications (PTMs), including acetylation and ubiquitination, dictate its stability and abundance (6–8).

Studies in the last decade have focused on the role of FGL1 in the liver, which is an organ with excellent regenerative ability and critical metabolic function. FGL1 may engage yet-to-be-defined receptors on hepatocytes, thereby influencing their proliferation and metabolic homeostasis. FGL1 acts as a hepatic growth factor that can promote hepatocyte proliferation and prevent hepatocyte apoptosis and oxidative stress when the liver is injured (9, 10). Excessive FGL1 causes hepatic lipid accumulation, inflammation and insulin resistance, leading to nonalcoholic fatty liver disease, obesity and type 2 diabetes (11–13).

Our attention is attracted by the immunoregulatory functions of FGL1, since FGL1 acts as a high-affinity ligand for the immune checkpoint receptor lymphocyte activation gene-3 (LAG-3), influencing the immune responses of T and natural killer (NK) cells (14, 15). Under physiological conditions, FGL1 restrains hepatic CD8^+^ T and NK cell activation and sustains regulatory T cell (Treg) numbers, preserving liver immune homeostasis (15). Under pathological conditions, its dysregulation promotes tumor immune escape in hepatocellular carcinoma (HCC), colorectal liver metastases, lung cancer, and gastric cancer (GC) (6, 8, 16–18). Moreover, it also modulates autoimmune diseases such as systemic lupus erythematosus (SLE), rheumatoid arthritis (RA) and primary Sjögren’s disease (pSjD), playing important regulatory roles beyond the liver (19–21). Consequently, therapeutic strategies such as monoclonal antibodies, blocking peptides or repurposed small-molecule drugs that interrupt FGL1-LAG-3 signaling or modulate FGL1 expression are emerging as powerful adjuncts to existing immunotherapies.

This review provides an overview of the structure, regulation, and binding molecules of FGL1, focuses on the immunoregulatory roles of FGL1 under physiological conditions and pathological conditions, and explores the potential of FGL1 as an immunotherapy target for disease.

## 2 The structure of FGL1

*FGL1* was originally cloned from the human HCC cDNA library in 1993 (2). It was initially named *hepatocyte-derived fibrinogen-related protein-1* (*HFREP-1*) on the basis of its structural characteristics and is also known as *hepassocin* (1, 2). *Liver fibrinogen-related gene-1* (*LFIRE-1*), which is highly homologous to *HFREP-1*, with a discrepancy in the 5’ untranslated region (5’UTR), was cloned from the human normal liver cDNA library. In the genomic sequence, the 5’UTR of *HFREP-1* cDNA is located 109 bp upstream of *LFIRE-1 (*22). *HFREP-1* and *LFIRE-1* encode the same fibrinogen-related protein and might be alternative splicing forms of the same gene. The *mouse fibrinogen-related protein-1* (*Mfrep-1*) gene is homologous to human *HFREP-1/LFIRE-1 (*23). In normal humans, FGL1 is expressed mainly in the liver, with lower levels in the pancreas and minimal expression elsewhere. In wild-type (WT) mice, FGL1 is expressed in the liver and brown adipose tissue, albeit at low levels in the latter (24). FGL1 is produced by the liver and is secreted throughout the body, with high levels of FGL1 protein present in the serum.

**Figure 1:**
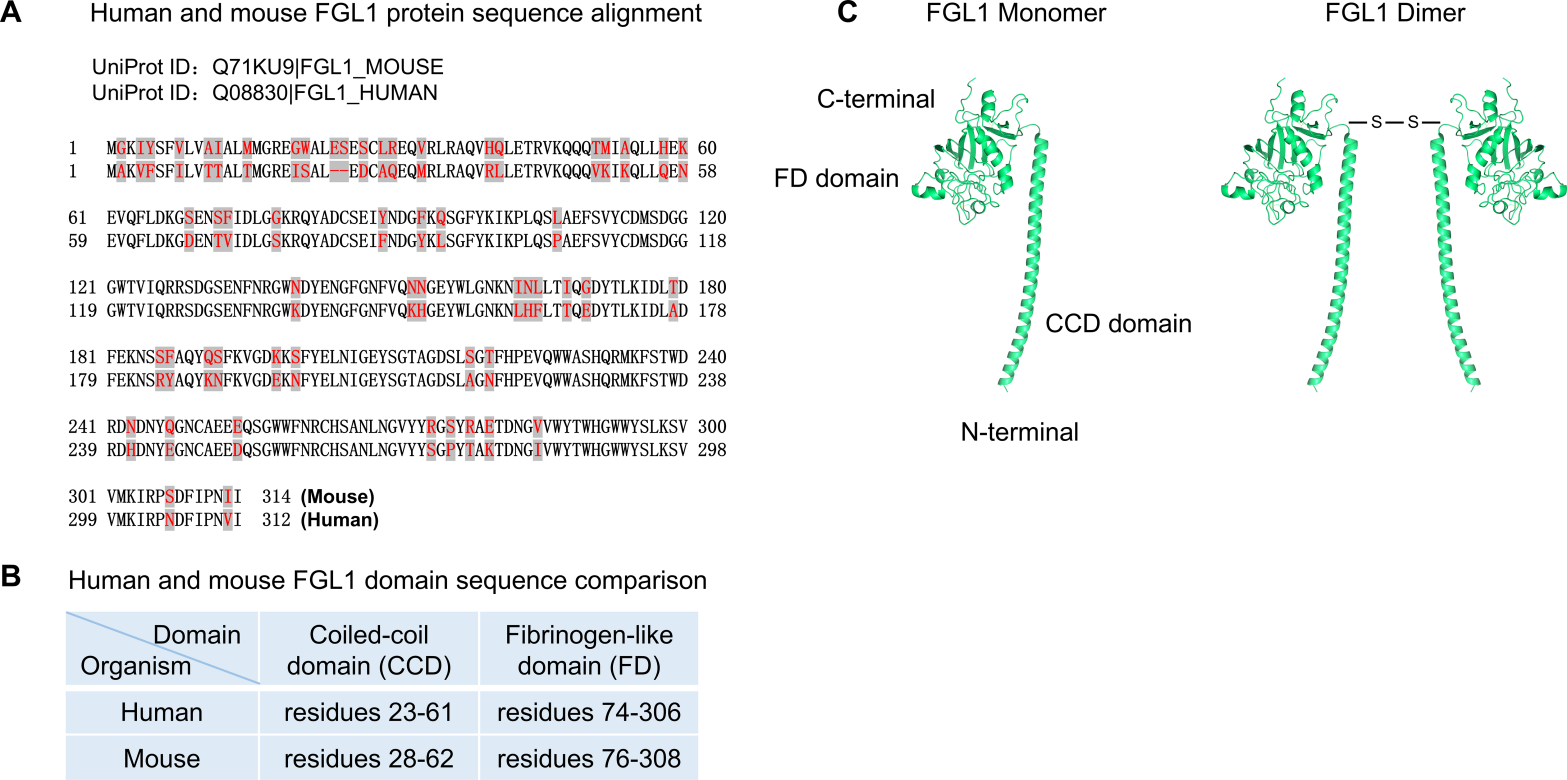
FGL1 sequence and structure. **(A)** Human and mouse FGL1 protein sequence alignment. **(B)** Human and mouse FGL1 domain sequence comparison. **(C)** Mouse FGL1 monomer structure prediction by AlphaFold. FGL1 homodimer connected by disulfide bonds between subunits. Available from: https://www.uniprot.org, with accession numbers including Q08830 (FGL1_HUMAN) and Q71KU9 (FGL1_MOUSE). AlphaFold Identifier: AF-Q71KU9-F1. https://alphafold.ebi.ac.uk/entry/Q71KU9. Data is available for academic and commercial use, under a CC-BY-4.0 license. AlphaFold Data Copyright (2022) DeepMind Technologies Limited.

The human *FGL1* gene is located on chromosome 8p22 and is composed of 10 exons and 9 introns, with a total length of approximately 31.0 kb. The human FGL1 protein precursor, which consists of 312 amino acids, contains a hydrophobic signal peptide sequence of 22 amino acids for protein secretion, a short N-terminal coiled-coil domain (CCD, residues 23–61 according to UniProt), and a conserved C-terminal fibrinogen-like domain (FD, residues 74-306) (25). Fibrinogen is composed of α, β and γ subunits. The mature human FGL1 protein shows 41.1% and 41.3% similarity to human fibrinogen β and γ subunits, respectively, and 47.9% similarity to mouse cytotoxic T lymphocyte-specific proteins (2). The mature human FGL1 protein has a molecular weight of 34.0 kDa and an isoelectric point (*pI*) of 5.45 (1). The recombinant human FGL1 protein typically exists as a homodimer connected by disulfide bonds between subunits, with a molecular weight of 68.0 kDa (26). According to UniProt, the mouse FGL1 protein is composed of 314 amino acids, exhibits approximately 82% similarity to human FGL1, and both FD domains are highly conserved (27). The recombinant mouse FGL1 protein mainly exists as an oligomer, with a small portion as a homodimer (**Figure 1**).

FGL1 has multiple potential phosphorylation sites, including four protein kinase C sites, seven casein kinase II sites, and one tyrosine kinase site (22). Although FGL1 lacks binding sites for platelets and thrombin, the plasma FGL1 protein can bind noncovalently to fibrin matrices in plasma clots, participating in coagulation. After coagulation, approximately 20% of FGL1 protein remains in the serum in a free form (1, 3).

## 3 The regulation of FGL1

In normal hepatocytes, the transcription of FGL1 is regulated primarily by transcription factors such as signal transducer and activator 3 (STAT3), hepatocyte nuclear factor-1α (HNF-1α), and CCAAT enhancer-binding protein β (C/EBPβ). Activation of the interleukin (IL)-6 receptor triggers the Janus kinase 2 (JAK2)-STAT3 pathway, after which phosphorylated STAT3 (p-STAT3) binds to the STAT3-binding site on the FGL1 promoter, which in turn enhances promoter activity and upregulates FGL1 expression (3, 4). High mobility group protein 1 and CREB-binding protein, which act as transcriptional coregulators, interact with HNF-1α in the cytoplasm first. These proteins subsequently translocate to the nucleus, where they, in conjunction with p-STAT3, initiate the transcription of FGL1 (4). Elevated glucose concentrations increase the activation of protein phosphatase 2A, which stimulates hepatic FGL1 expression through the HNF-1α and STAT3 pathways (28). Moreover, palmitate-induced endoplasmic reticulum stress activates p38 mitogen-activated protein kinase (p38 MAPK), thereby facilitating C/EBPβ binding to the FGL1 promoter and driving FGL1 transcription (5). The FGL1 promoter contains two hypoxia-inducible transcription factor-binding sites that induce FGL1 expression under hypoxic conditions (29).

Protein PTMs are essential for regulating protein structure, activity, and function. FGL1 undergoes several direct PTMs, such as acetylation and ubiquitination. Acetylation at lysine 98 of FGL1 facilitates its ubiquitination and degradation through the ubiquitin-proteasome pathway. Sirtuin 2 (SIRT2), a deacetylase, reduces FGL1 acetylation, thereby maintaining its stability (6). E3 ubiquitin ligase F-box protein 38 (FBXO38) mediates ubiquitination at lysine 48 of FGL1, promoting its degradation (7). Nuclear factor-kappa B (NF-κB) signaling upregulates OTU deubiquitinase 1 (OTUD1), which decreases FGL1 ubiquitination and enhances its stability (8).

Moreover, in HepG2 and Huh7 cells, ubiquitin-specific peptidase 7 (USP7) upregulates FGL1 by deubiquitinating the transcription factor PR domain zinc finger protein 1 (PRDM1), which transcriptionally activates FGL1 (30). Additionally, protein arginine methyltransferase 5 (PRMT5)-catalyzed symmetric dimethylation of transcription factor 12 (TCF12) at arginine 554 (R554) promotes TCF12 binding to the FGL1 promoter region, which transcriptionally activates FGL1 in liver cancer cells (31). In Kirsten rat sarcoma viral oncogene homologue (*KRAS*)-driven lung adenocarcinoma (LUAD), *KRAS* modulates the methylation of Yes-associated protein (Yap) through the ERK1/2-SET1 histone methyltransferase (SET1A) pathway, promoting Yap-induced transcription of FGL1 in LUAD cells (16). Overall, aberrant PTMs of FGL1 are implicated in several malignancies.

Mature FGL1 is secreted extracellularly and functions via autocrine or paracrine mechanisms. Currently, there is insufficient evidence for the presence of stable FGL1 on the membrane surface of hepatocytes or other tumor cells.

## 4 Binding molecules and signaling pathways of FGL1

FGL1 exerts various biological activities by binding to different molecules. To date, three molecules involved in different signaling pathways have been identified.

### 4.1 LAG-3

In 2019, Jun Wang et al. discovered for the first time that FGL1 could specifically bind to the inhibitory receptor LAG-3, revealing its immunological function (14). LAG-3 is expressed primarily on activated CD8^+^ T cells, CD4^+^ T cells, Tregs, NKT cells, NK cells, and plasmacytoid dendritic cells (pDCs), with minimal expression in myeloid cell populations (32).

LAG-3, a type I transmembrane protein, is composed of an extracellular region, a transmembrane region, and an intracellular region. The extracellular region of LAG-3 contains four immunoglobulin (Ig)-like domains (D1 to D4), similar to CD4 (33). LAG-3 interacts with its ligands through distinct domains. Specifically, a loop structure within the D1 domain (loop 1, approximately 30 amino acids) binds to major histocompatibility complex class II (MHCII). LAG-3 selectively recognizes the conformationally stable peptide-MHCII (pMHCII) complex (34). LAG-3 forms cis-homodimers through a conserved hydrophobic structure within the D2 domain of its extracellular region, which is crucial for its suppressive activity (35). FGL1 binds to another loop structure (loop 2) within the D1 domain of the extracellular region of LAG-3 through its FD structure, promoting the formation of higher-order oligomers from LAG-3 homodimers on the cell surface. Loops 1 and 2 within the D1 domain of LAG-3 exhibit partially overlapping binding surfaces. This is consistent with preclinical evidence showing that antibodies such as relatlimab and 15011 can block LAG-3 interactions with both FGL1 and MHCII (36). Physiological concentrations of FGL1 (10 nM) can induce the formation of higher-order oligomers from LAG-3 dimers. However, high concentrations of FGL1 (100 nM) do not effectively promote the oligomerization of LAG-3 homodimers, and the underlying mechanism remains unclear; this may be related to the spatial structure of the protein (36). Beyond canonical ligands, galectin-3 and liver sinusoidal endothelial cell lectin (LSECtin) have been suggested to interact with glycans on LAG-3 (**Table 1**).

**Table 1:** Major immunosuppressive ligands of LAG-3.

Ligand	Cellular sources	Binding domain(s) on LAG-3	Disease relevance and functional outcomes
FGL1	• Hepatocytes • Tumor cells (lung cancer, melanoma, CRC, etc.)	D1-D2 domain (partially overlapping with MHCII binding site) • Core region: D1 loop 2	• Physiological conditions: Maintains hepatic immune homeostasis • Tumors: Inhibits T cell activation and promotes T cell exhaustion (synergizes with PD-1) • Autoimmune diseases: Elevated in RA, SLE-LD and pSjD; regulates naïve/memory T cell homeostasis and Treg function (role remains controversial) • Transplantation: Attenuation of FGL1-LAG-3 signaling promotes immune rejection
MHCII (stable pMHCII)	• Professional APCs (DCs, B cells, and macrophages) • Tumor cells • Non-hematopoietic cells under inflammatory conditions (e.g., IFN-γ-induced epithelial cells)	D1 domain • Core region: D1 loop 1 (additional contact with D2 domain for stable interaction)	• Autoimmune diseases: High-affinity pMHCII-LAG-3 interaction delivers inhibitory signals to autoreactive T cells; implicated in T1D and MS pathogenesis • Tumors: Inhibits T cell activation and promotes tumor immune escape (synergizes with PD-1) • Infection: Sustains T cell exhaustion in chronic infections
Galectin-3	• Tumor cells (breast cancer, melanoma, CRC, etc.) • Stromal cells • Immune cells	• N-glycan chains on LAG-3 at glycosylation sites within the D1-D2 interdomain region (distinct from the MHCII binding site)	• Tumors: Galectin-3-LAG-3 axis suppresses TCR signaling and IL-2 production; associated with poor prognosis in solid tumors
LSECtin	• LSECs	• N-linked glycans on LAG-3 (distinct from the MHCII binding site)	• Hepatic tolerance: Maintenance of liver immune homeostasis • Tumors: Inhibits T cell activation • Liver cirrhosis: Attenuates Th17 differentiation

FGL1, fibrinogen-like protein 1; MHCII, major histocompatibility complex class II; pMHCII, peptide-MHCII complex; LSECtin, liver sinusoidal endothelial cell lectin; CRC, colorectal cancer; APC, antigen-presenting cell; DC, dendritic cell; RA, rheumatoid arthritis; SLE-LD, systemic lupus erythematosus and liver damage; pSjD, primary Sjögren’s disease; T1D, type 1 diabetes; MS, multiple sclerosis; TCR, T cell receptor; Th17, T helper cell 17.

LAG-3 engages diverse ligands in a context-dependent manner; MHCII may predominate in lymphoid tissues and autoimmune contexts, whereas FGL1 may play more prominent roles in the liver and MHCII-deficient tumor microenvironment. Importantly, multiple ligands can coexist within the same niche, establishing redundant or synergistic inhibitory axes (**Table 1**). Although either FGL1 deficiency or LAG-3 deficiency exhibits comparable antitumor effects in mouse models, this does not rule out the existence of additional functional receptors for FGL1 (14). Indeed, Maruhashi et al. reported that stable pMHCII, rather than FGL1, serves as the functional ligand of LAG-3 that suppresses T cells in autoimmunity and antitumor immunity, but their study does not completely exclude the possibility that the FGL1-LAG-3 interaction is required for the stable pMHCII-mediated suppression of antitumor immunity by LAG-3 (34). Notably, the detection of robust FGL1 binding to LAG-3-deficient T cells suggests that FGL1 may associate with molecule(s) other than LAG-3 to suppress T cells. Further analyses are expected to reveal the actual role of FGL1 in regulating T cell function.

LAG-3 lacks classical inhibitory motifs, and its inhibitory function relies on three evolutionarily conserved intracellular motifs, the FSALE motif, the KIEELE motif, and the C-terminal EP-repeat motif. The functions of these domains remain to be fully defined. Upon binding to pMHCII, the FSALE and EP motifs in the intracellular region of LAG-3 transmit two distinct inhibitory signals that suppress T cell activation (37). The KIEELE motif is essential for the expansion and activation of effector T cells and for hybridoma T cells to produce IL-2 (38, 39). LAG-3 migrates to immunological synapses upon activation of CD4^+^ T or CD8^+^ T cells and is positioned adjacent to the TCR-CD3 complex. Its EP motif lowers the local pH at the synapse, causing CD4 or CD8 coreceptor molecules to dissociate from lymphocyte-specific protein tyrosine kinase, thereby inhibiting TCR signaling (40). This process can occur ligand-independently. Nevertheless, evidence shows that the critical role of ligand engagement in facilitating LAG-3-mediated inhibitory function depends on the cytoplasmic FSALE motif (41). The interaction of MHCII or membrane-bound FGL1 (not soluble FGL1) with antigen-presenting cell-mediated ligand engagement triggers rapid LAG-3 ubiquitination, releasing the LAG-3 signaling tail, particularly the FSALE motif, from its association with the cell membrane, consequently unleashing its inhibitory function (42). As a soluble ligand, FGL1 promotes the oligomerization of LAG-3 dimers, inhibiting T lymphocyte proliferation and the secretion of cytokines such as IL-2, interferon-γ (IFN-γ) and tumor necrosis factor-α (TNF-α) (14, 36). The exact mechanisms by which FGL1 affects the intracellular signaling pathways of LAG-3 are still under investigation.

### 4.2 Bone morphogenetic protein 6

Hepcidin is synthesized and secreted by the liver and plays a crucial role in maintaining iron metabolic balance by regulating the absorption, distribution, and utilization of iron. Recently, FGL1 was shown to be a potent suppressor of hepcidin *in vitro* and *in vivo*. FGL1 directly binds to bone morphogenetic protein 6 (BMP6), thereby inhibiting the canonical BMP-SMAD signaling cascade to repress hepcidin transcription. Moreover, the FD domain in the C-terminus of FGL1 is responsible for hepcidin suppression, whereas the N-terminal domain is inactive (29).

### 4.3 Activin receptor-like kinase 5

Activin receptor-like kinase 5 (ALK5), also known as transforming growth factor-β receptor type I (TGFβR1), is a key receptor of the TGFβ signaling pathway. FGL1 binds to ALK5 on the surface of renal tubular epithelial cells with high affinity, thereby preventing ALK5 ubiquitination and potentiating TGF-β/SMAD signaling to promote renal fibrotic progression. The FD domain (150–312 amino acid region) of FGL1 could interact with ALK5, while the 1–150 amino acid region could not (43). Besides epithelial cells and endothelial cells, ALK5 is also expressed on macrophages and T cells, playing important roles in macrophage inflammation and T cell trafficking (44, 45). It remains unknown whether FGL1 engages ALK5 on these cells to exert immunoregulatory functions.

### 4.4 Potential binding molecule(s)

In addition to LAG-3 and BMP6, FGL1 may have other potential binding molecule(s). Maruhashi et al. reported that the pentameric domain of cartilage oligomeric matrix protein (Comp-FGL1) strongly binds to LAG-3-deficient T cells *in vitro*, indicating the existence of FGL1-binding molecule(s) other than LAG-3 on activated T cells (34). High concentrations of FGL1 have no effect on the oligomerization of LAG-3 homodimers and may regulate T cells in other ways. Meng-Meng Cao et al. demonstrated that FGL1-binding molecule(s) are present on the surface of human L02 liver cells by coincubating fluorescein-labelled recombinant human FGL1 protein with human L02 liver cells. However, the molecules that interact with FGL1 have not yet been clearly identified. FGL1 interacts with binding molecule(s) in an autocrine and dose-dependent manner, promoting the proliferation of human L02 liver cells (46).

FGL1 has also been shown to induce the phosphorylation of the tyrosine protein kinase Src and then transactivate epidermal growth factor receptor (EGFR) and downstream extracellular signal-regulated kinase (ERK) 1/2 in a ligand-independent manner, promoting the proliferation of normal liver cells (47). In addition, FGL1 can promote hepatic lipid accumulation and insulin resistance (IR) via the ERK1/2 pathway (11, 13). In addition to regulating hepatocytes, liver-derived FGL1 promotes the phosphorylation of c-Jun N-terminal kinase in skeletal muscle cells through the EGFR pathway. This impairs the insulin sensitivity of skeletal muscle cells, leading to IR (5). FGL1, which is also weakly expressed in adipose tissue, can promote lipid synthesis in the 3T3-L1 mouse embryonic fibroblast (preadipocyte) cell line in a manner dependent on ERK1/2-C/EBPβ (12).

**Figure 2:**
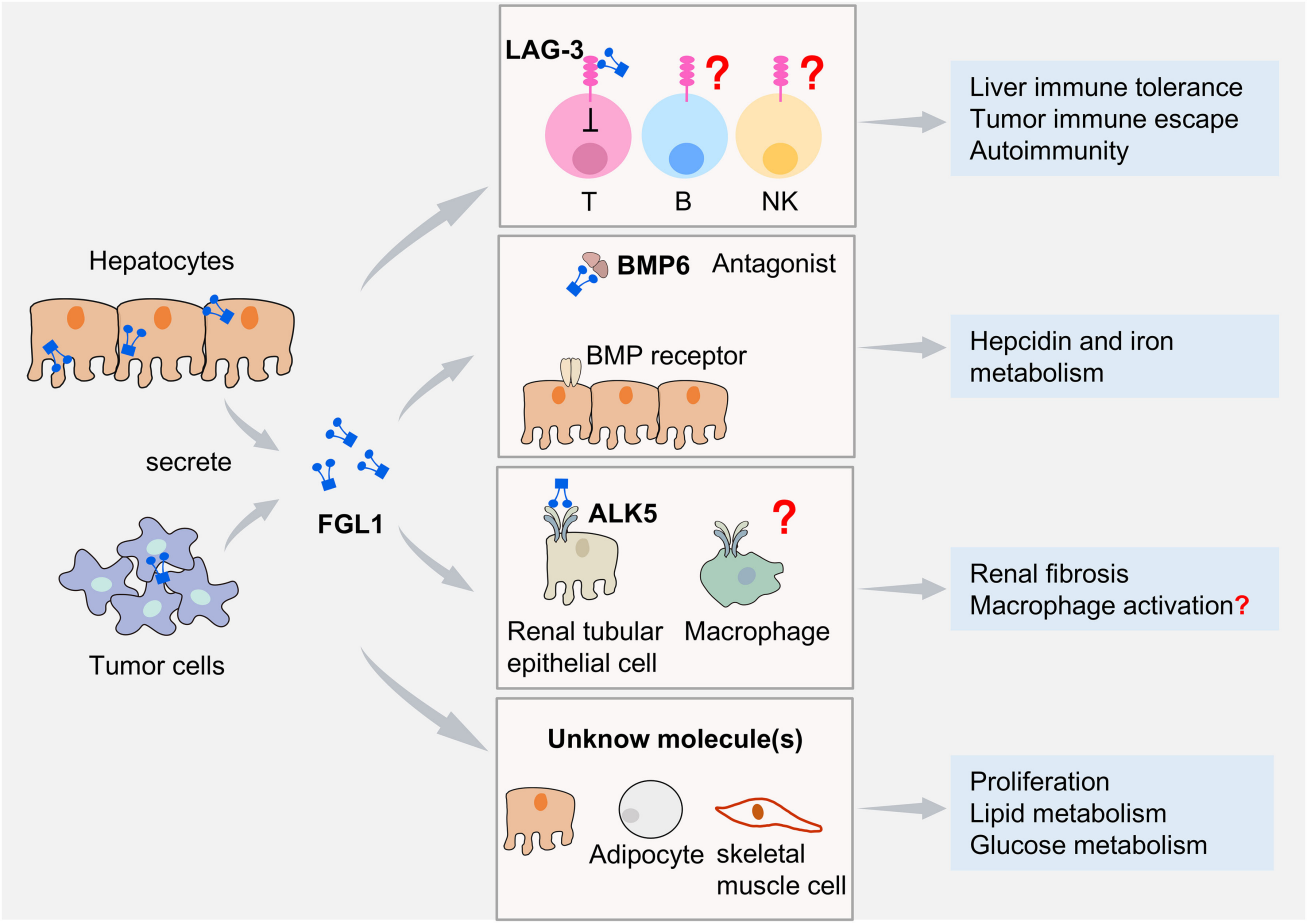
FGL1 and its binding molecules. FGL1 is expressed mainly by hepatocytes and additionally in certain tumor cells. FGL1 mediates diverse biological effects by binding to distinct molecules. Its specific interaction with the inhibitory receptor LAG-3 suppresses T cell function. B cell and NK cell functions are also affected by FGL1, but it remains uncertain whether this effect is completely dependent on LAG-3. FGL1 binding to BMP6 antagonizes BMP6−SMAD signaling, inhibiting iron regulatory protein expression and modulating iron metabolism. FGL1 interacts with ALK5 to enhance the TGF-β/SMAD signaling pathway in renal tubular epithelial cells and promote renal fibrosis progression. It remains unknown whether FGL1 regulates immune cells via ALK5. Through engagement with unidentified molecules, FGL1 promotes hepatocyte proliferation and regulates lipid metabolism in hepatocytes and adipocyte, as well as glucose metabolism in skeletal muscle cells.

Overall, FGL1 performs immunoregulatory functions mainly via its receptor LAG-3 and may be involved in physiological and pathological processes, such as tumor immune escape and autoimmune diseases. Other receptors, such as ALK5, are also expressed in immune cells such as macrophages and T cells, but whether FGL1 regulates these cells via ALK5 remains unclear. FGL1 may act on different cell types through different receptors, and the existence of additional receptors requires further in-depth investigation (**Figure 2**).

## 5 The immunoregulatory roles of FGL1 under physiological conditions

FGL1 was initially found to be specific to liver and HCC cell lines (2). We further demonstrated that FGL1 is specifically expressed by hepatocytes but not nonparenchymal cells, including liver-infiltrating lymphocytes such as hepatic CD8^+^ T and NK cells, in WT mice under physiological conditions (15). Moreover, hepatic lymphocytes express the receptor LAG-3, indicating that FGL1 has immunoregulatory effects on these cells.

*Fgl1*-deficient mice have an overall normal appearance, organ size, and number of litters, indicating that FGL1 does not affect the development or growth of mice globally. However, at 14~16 months of age, compared with WT mice, 5/8 female but not male mice presented elevated levels of anti-double-stranded DNA autoantibodies in their plasma. There are small but significant increases in central memory-like CD8^+^ T cell subsets and decreases in two B-cell subsets (14). Furthermore, our study revealed that compared with WT mice, hepatic CD8^+^ T and NK cells showed significantly increased numbers and higher activation levels in *Fgl1* deficient mice (8~10 w), as well as decreased numbers of Tregs, suggesting that FGL1 plays specific regulatory roles in the liver under physiological conditions (15). These findings indicate that FGL1 may participate in regulating autoimmunity and immune homeostasis.

## 6 The immunoregulatory roles of FGL1 in pathological conditions

### 6.1 Primary liver cancer and liver metastasis

Previous studies revealed that FGL1 is frequently downregulated in HCC because of allelic loss. A reduction in or deficiency of FGL1 enhances HCC proliferation and colony formation and is significantly associated with the degree of tumor differentiation (22). Furthermore, the activation of protein kinase B (PKB/AKT) and its downstream targets of mammalian target of rapamycin (mTOR) signals increases in *Fgl1-*deficient mice, significantly increasing the incidence of HCC in response to treatment with the chemical carcinogens diethylnitrosamine and low-dose phenobarbital (48). FGL1 may reduce the incidence of HCC by regulating hepatocyte proliferation, survival and metabolism. Exogenous supplementation with recombinant FGL1 protein has no significant effect on the proliferation of HCC tumor cells, suggesting that the intracellular and extracellular effects of FGL1 differ (46).

Since FGL1 has been identified as a major immune inhibitory ligand of LAG-3, its role in tumors has been further investigated. Aberrant transcriptional activation and PTMs lead to elevated FGL1 expression. Studies have shown that high expression levels of FGL1 are significantly associated with poor overall survival in HCC patients (6). FGL1 in HCC tissues is relatively stable, and the FGL1-LAG-3 interaction increases, thus suppressing the antitumor immunity of CD8^+^ T cells and promoting the development of HCC (30, 31). Increased FGL1-LAG-3 interaction in CD8^+^ tissue-resident memory T cells (T_RM_) in end-stage HCC may result in CD8^+^ T_RM_ cell exhaustion, causing tumor immune escape (6, 49).

In addition to being highly susceptible to primary tumors, the liver is also a common site of tumor metastasis, including a wide variety of malignancies, such as colorectal cancer (CRC), melanoma, lung cancer and breast cancer (50). Compared with hepatocytes, tumor cells, including Hepa1-6, MC38 and B16-F10 cells, express lower levels of *Fgl1* mRNA and FGL1 protein *in vitro* (15). Jia-Jun Li et al. reported that FGL1 protein expression in MC38 cells was distinctly elevated in liver metastatic tumor cells compared with cecum termini, suggesting that FGL1 expression is different *in vivo* and *in vitro* (8). Although CRC tumor cells express FGL1, our previous study revealed that FGL1 deficiency in mice inhibited CRC liver metastasis and prolonged survival. FGL1 blockade promoted the antitumor activities of CD8^+^ T and NK cells, confirming that hepatocyte-derived FGL1 negatively regulates liver metastasis to a certain extent (15). On the other hand, CRC tumor cell-derived FGL1 facilitates the progression of liver metastasis. Mechanistically, tumor-associated macrophages activate the NF-ĸB pathway by secreting TNF-α and IL-1β in the liver microenvironment and transcriptionally upregulate OTUD1 expression in CRC tumor cells, which enhances FGL1 stability via deubiquitination, promoting CRC liver metastasis (8). Clinically, high plasma FGL1 levels predict poor outcomes, and reduced immune checkpoint blockade therapy benefits CRC patients with liver metastasis (8). Both hepatocyte-derived FGL1 and tumor cell-derived FGL1 promote liver metastasis, indicating that targeting FGL1 may be a new strategy for liver metastasis.

### 6.2 Other tumors

In addition to its role in liver cancer, FGL1 is involved in the development of several tumors, including lung cancer, melanoma, urothelial carcinoma (UC), stomach adenocarcinoma (STAD), GC and adrenocortical carcinoma (ACC).

The role of FGL1 in cancer is likely contextual. On the one hand, FGL1 plays vital immunoregulatory roles in the tumor environment. FGL1 promotes the secretion of IL-2 and TNF-α by T cells *in vitro*, simultaneously inducing their apoptosis, suggesting that FGL1 may promote the activation-induced apoptosis of T cells in LUAD (51). Moreover, FGL1 inhibits CD8^+^ T-cell infiltration into LUAD tumors and their ability to release IFN-γ, TNF-α and perforin, promoting immune escape in LUAD (16, 52). Elevated FGL1 levels are associated with worse overall survival (OS) in patients with lung cancer, metastatic melanoma, STAD and GC (14, 53, 54). Elevated LAG-3 expression, particularly in conjunction with FGL1 coexpression, is associated with reduced responsiveness to PD-(L)1 blockade therapy and unfavorable oncological outcomes in advanced urothelial carcinoma (UC) (18). FGL1 is also upregulated in microsatellite instability (MSI)-type GC cells and can bind to LAG-3 on CD8^+^ T cells. This binding inhibits the activity of CD8^+^ T cells, rendering them unable to release cytotoxic substances such as IFN-γ and TNF-α and effectively killing tumor cells (17). Targeting FGL1 facilitates anticancer immunity against MSI-high GC.

On the other hand, FGL1 is involved in the proliferation, migration and invasion of tumor cells. For example, FGL1 is highly expressed in the cytoplasm of LUAD cells and affects LUAD proliferation by regulating *MYC* target genes and myosin heavy chain 9 (MYH9) (51, 55). FGL1 promotes the proliferation, invasion, migration, and epithelial−mesenchymal transition (EMT) of GC cells (54). Elevated expression of FGL1, which physically interacts with centromere protein M, may regulate the migration and invasion of ACC cells, potentially by influencing collagen organization and cell adhesion (56).

The roles of FGL1 in the tumor environment are complex, and dual immunological and tumor biological regulation by FGL1 should be considered when FGL1 is targeted for tumor therapy.

### 6.3 Autoimmune diseases

FGL1 is upregulated in patients with SLE and liver damage (SLE-LD), RA and pSjD, playing important roles in the crosstalk between the liver and other organs and maintaining the balance of immunity and tolerance (20, 21).

The concentration of FGL1 is closely related to the serum IL-6 level in patients with autoimmune diseases. In patients with SLE-LD, IL-6 produced by hepatocytes acts on hepatocytes themselves, further promoting the production of FGL1. In the pSjD mouse model, the serum levels of FGL1 are significantly increased due to increased activation of IL-6 signaling in CD4^+^ T cells of the salivary glands and spleen. Higher expression of the FGL1 receptor LAG-3 is detected in Tregs from patients with SLE-LD. The FGL1-LAG-3 signaling axis inhibits Treg proliferation and impairs the suppressive activity of Tregs by limiting IL-10 secretion. Furthermore, FGL1-LAG-3 signaling promotes the production of pathogenic IL-17A and IL-21 by CD4^+^ T cells in patients with SLE. High levels of FGL1 aggravate disease in patients with SLE-LD (19). However, FGL1 may control the onset of autoimmune pathology in the target organ of pSjD model mice by regulating naïve/memory T cell homeostasis after T-cell activation such that *Fgl1-*deficient mice exhibit more severe pathological lesions (21).

In a collagen-induced arthritis (CIA) mouse model, FGL1 recombinant protein administered intraperitoneally significantly attenuated T cell-mediated autoimmune disease-related arthritis (57). Moreover, FGL1-overexpressing exosomes (FGL1-Exos) effectively suppressed inflammation scores, joint destruction, and the inflammatory response in an RA rat model (58). The increased levels of FGL1 in patients with RA may be related to liver inflammation or injury. Thus, targeting FGL1 may be a therapeutic strategy for RA, and more experimental basis is needed.

FGL1 has distinct regulatory effects on different immune cell subsets, which may be related to the expression levels of FGL1 and its receptor LAG-3. The outcome of its regulation of Tregs and effector T cell functions determines the development and progression of autoimmune diseases.

### 6.4 Organ transplantation

Notably, the liver is a unique immune-tolerant organ. Compared with other organ transplants, liver transplants result in minimal rejection. Moreover, graft rejection is significantly reduced if the recipient undergoes a liver transplant from the same donor prior to receiving another organ transplant. The interaction of hepatocytes and immune cells plays an important role in inducing liver transplant tolerance. FGL1 is specifically expressed by hepatocytes, and its expression level is significantly increased after partial hepatectomy (23). T cell-mediated cellular immunity plays an important role in transplant rejection, so it can be hypothesized that FGL1-mediated suppression of T cell function may play a role in liver transplantation.

In a heart allograft model, small extracellular vesicles expressing FGL1 and programmed death ligand 1 (PD-L1) could reestablish immune tolerance and significantly alleviate immune rejection (59). With the occurrence of transplantation rejection, the expression levels of LAG-3 and PD-1 increase significantly in T cells, suggesting the activation of these T cells. However, neither the PD-L1 nor FGL1, was increased in the spleens of transplanted mice; thus, enhancing these inhibitory signals may alleviate immune rejection. Targeting FGL1 may be a therapeutic strategy to reduce immune rejection in organ transplantation.

## 7 Targeting FGL1 for disease treatment

Recently, preclinical experiments that target FGL1 have been conducted to treat various diseases, such as tumors, liver injury and RA. These reagents primarily include monoclonal antibodies against FGL1 (anti-FGL1 mAb), peptides that block FGL1, clinical drugs or inhibitors that inhibit FGL1 expression, and recombinant human/mouse FGL1 protein (**Table 2**).

**Table 2:** Preclinical data on FGL1 targeting for disease therapy.

Monoclonal antibody				
Drugs	Year	Type of disease	Mouse models	Reference
Anti-FGL1 mAb	2019	CRC	MC38 subcutaneous tumor model	(14)
Anti-FGL1 mAb	2019	HCC	Hepa1–6 subcutaneous tumor model	(14)
Anti-FGL1 mAb	2023	CRC	MC38 subcutaneous tumor model	(7)
Anti-FGL1 mAb	2024	HCC	Hepa1–6 orthotopic tumor model	(15)
Anti-FGL1 mAb	2024	Liver metastasis	MC38 and B16-F10 liver metastasis model	(15)
Anti-FGL1 mAb	2024	LUAD	LLC subcutaneous tumor model	(16)
Anti-FGL1 mAb	2025	GC	HGC-27 subcutaneous immune reconstituted MSI GC mouse model	(17)
Recombinant protein				
Drugs	Year	Type of disease	Animal models	Reference
Recombinant human FGL1 protein	2010	Fulminant hepatic failure	D-galactose (D-Gal)- or carbon tetrachloride (CCl4)-induced liver injury	(9)
Recombinant human FGL1 protein	2021	Acute liver injury	D-Gal- or CCl4-induced liver injury	(10)
Recombinant mouse FGL1 protein	2021	RA	Collagen-induced arthritis	(57)
Clinical drugs				
Drugs	Year	Type of disease	Mouse models	Reference
Oxysophocarpine	2020	HCC	Hepa1–6 subcutaneous tumor model	(65)
Aspirin	2023	HCC	Hepa1–6 subcutaneous tumor model	(6)
Benzethonium chloride	2023	Liver metastasis	MC38 liver metastasis model	(8)
vorinostat (SAHA)	2024	LUAD	LLC subcutaneous tumor model	(52)
Others				
Drugs	Year	Type of disease	Mouse models	Reference
Blocking peptide	2024	CRC	MC38 subcutaneous tumor model	(60)
inhibitor of USP7	2024	HCC	Hepa1–6 subcutaneous tumor model	(30)
PRMT5 methyltransferase inhibitor GSK591	2025	HCC	Hepa1–6 subcutaneous tumor model	(31)
LAG-3 released by nanoengineered T cell membrane-coated nanodecoy	2025	Breast cancer Lung metastasis	4T1 subcutaneous tumor model and lung metastasis mice model	(66)
P-hydroxybenzaldehyde (PHBA)	2026	Chronic kidney disease	Unilateral ureteral obstruction	(43)

CRC, colorectal cancer; HCC, hepatocellular carcinoma; LUAD, lung adenocarcinoma; GC, gastric cancer; RA, rheumatoid arthritis; MSI, microsatellite instability.

Treatment with an anti-FGL1 mAb directly inhibits the interaction between FGL1 and LAG-3, enhancing the antitumor immunity of CD8^+^ T and NK cells in CRC liver metastasis (15). Dual blockade of PD-(L)1 and FGL1 further increased the therapeutic efficacy of anti-PD-(L)1 immunotherapy in CRC and lung cancer, supporting treatment with dual blocking antibodies (14, 16). Immune-related adverse events, such as skin toxicity, colitis, hepatitis, pneumonia, nephritis and endocrine diseases, cannot be ignored in immunotherapy. Low-dose anti-FGL1 mAb treatment several times did not cause hepatocyte injury in the mice. However, given the established role of FGL1 in maintaining hepatic immune tolerance and that its deficiency increases the risk of dermatitis in older mice, long-term or high-dose use of FGL1 blockade antibodies may carry risks related to the disruption of liver immune homeostasis and the induction of systemic autoimmunity, particularly in older individuals. Clinical trials are needed to prove the safety of FGL1 blockade. In addition to antibodies, blocking peptides show great potential due to several advantages, such as better tumor penetration and lower cost. Peptides blocking the LAG-3-FGL1 interaction can restore T cell function, and bispecific peptides targeting both PD-1-PD-L1 and LAG-3-FGL1 synergize with radiotherapy to further enhance the antitumor immune response (60). Radiation-induced increases in FGL1 expression in mouse livers and lungs indicate that tumor radiotherapy may result in immunosuppression (61, 62). Thus, combining radiotherapy with blocking antibodies or peptides might enhance therapeutic efficacy.

Clinical drugs such as aspirin and benzethonium chloride promote the degradation of FGL1 by regulating the PTM process, thereby inhibiting liver cancer progression and enhancing immunotherapy efficacy (6, 8). Compared with untreated controls, mice with HCC treated with aspirin in combination with an anti-PD-L1 blockade antibody exhibited significantly improved survival (6). PD-1 blockade alone had no significant benefit in a CRC liver metastasis mouse model; however, benzethonium chloride alone significantly slowed the progression of liver metastasis and enhanced the efficacy of PD-1 blockade when combined (8). Research has shown a time-and dose-dependent reduction in HCC risk with aspirin use and a significant decrease in HCC-and liver-related mortality (63, 64). This provides a strong basis for evaluating alternatives to cancer immunotherapy, and blocking antibodies may be more efficacious in combination with certain drugs. The efficacy and side effects of these clinical drugs in the treatment of liver cancer and other cancers still need to be evaluated.

Recombinant human or mouse FGL1 protein promotes the proliferation of hepatocytes to protect the liver from injury; on the other hand, it inhibits inflammation caused by T-cell activation to prevent the occurrence of autoimmune diseases (48). Targeting FGL1 may be an effective therapeutic strategy for liver injury complicated by inflammation.

Overall, FGL1 performs important immunoregulatory functions under both physiological and pathological conditions; consequently, targeting key molecules in the regulatory pathways of FGL1 expression or targeting its receptor constitutes a highly promising therapeutic strategy for diverse diseases. Currently, FGL1-targeted therapeutic strategies remain predominantly confined to preclinical animal models, with clinical translational validation markedly inadequate. Future clinical studies are therefore warranted to optimize dosing regimens, treatment duration, and therapeutic windows tailored to distinct disease contexts, while concurrently monitoring hepatic transaminases and autoantibody levels to prevent breaching autoimmune activation thresholds or inadvertently inducing immunosuppression.

## 8 Conclusions and perspectives

FGL1, a hepatocyte-secreted fibrinogen-like protein, has emerged as a pivotal regulator of immunity. Its structure and function have been preliminarily elucidated. Structurally, it contains a signal peptide, a coiled-coil domain, and a conserved C-terminal fibrinogen-like domain that mediates receptor engagement. Transcriptionally, its expression is driven by STAT3, HNF-1α, and C/EBPβ and is fine-tuned posttranslationally by acetylation and ubiquitination. Hepatocytes cope with harsh environments, such as inflammation, hypoxia, and high glucose, by precisely regulating FGL1 expression. FGL1 functions through three validated axes. First, it binds LAG-3 on T cells, triggering inhibitory motifs that suppress IL-2, IFN-γ and TNF-α production and promote T-cell exhaustion. Second, it antagonizes BMP6, blunts SMAD-mediated hepcidin transcription and modulates systemic iron homeostasis. Third, it binds ALK5 on renal tubular epithelial cells, promoting fibrotic progression by potentiating TGF-β signaling.

In oncology, FGL1 is a double-edged sword. Loss of FGL1 alleles in HCC activates PI3K/AKT/mTOR signaling and accelerates carcinogenesis; however, during tumor progression, malignant cells and hepatocytes may upregulate FGL1 expression through compensatory mechanisms, transcriptional activation, or PTMs. The ensuing FGL1-LAG-3 interaction creates an immunosuppressive niche that promotes primary HCC and liver metastases of CRC, melanoma and GC. Clinically, high plasma FGL1 levels predict poor overall survival and resistance to PD-(L)1 blockade, identifying FGL1 as a companion biomarker and a rational target for combination immunotherapy.

Conversely, in autoimmune settings, FGL1 deficiency may unleash autoreactive T and B cells to promote the development of disease. Exogenous FGL1- or FGL1-loaded extracellular vesicles suppress inflammation in a CIA mouse model or an RA rat model, illustrating their therapeutic potential as immunosuppressive drugs. However, studies have shown that excessive FGL1 also accelerates disease progression. Thus, FGL1 plays an important role in maintaining immune balance.

The following factors are worth considering. (1) Mechanistic clarity: The structural resolution of the FGL1-LAG-3, FGL1-BMP6, FGL1-ALK5 complexes will guide rational drug design and reveal whether additional receptors exist. (2) Precision targeting: Bispecific antibodies or nanobodies that block FGL1 in tumors but spare or even enhance its activity in autoimmune tissues minimize on-target toxicity. (3) Combinatorial strategies: Integrating blockade of FGL1-LAG-3 agents with radiotherapy or metabolic modulators could overcome adaptive resistance and extend benefits to solid tumors beyond the liver. Ultimately, FGL1 exemplifies how a single secreted protein can orchestrate immunity across health and disease. Deciphering its context-dependent biology and developing selective modulators could lead to the transformation of both cancer immunotherapy and immune-mediated disorders.
